# (Un)Covering the COVID-19 Pandemic: Framing Analysis of the Crisis in Canada

**DOI:** 10.1017/S0008423920000372

**Published:** 2020-04-29

**Authors:** William Poirier, Catherine Ouellet, Marc-Antoine Rancourt, Justine Béchard, Yannick Dufresne

**Affiliations:** 1 Département de science politique, Université Laval, 1030, avenue des Sciences-Humaines, Québec (Québec) G1V 0A6; 2 Department of Political Science, University of Toronto, Sidney Smith Hall, 100 St. George Street Toronto (Ontario) M5S 3G3

## Abstract

The current COVID-19 crisis is unprecedented in recent history. On April 1, 2020, the Secretary-General of the United Nations, Antonio Guterres, warned that the world was facing the most challenging crisis since World War II (Associated Press, 2020). With the pandemic taking on an unprecedented magnitude in the twenty-first century, it quickly monopolized media attention. As of early April, Radar+'s large dataset showed that about 65 per cent of headlines on major Canadian media websites were related to the COVID-19 pandemic.

The current COVID-19 crisis is unprecedented in recent history. On April 1, 2020, the Secretary-General of the United Nations, Antonio Guterres, warned that the world was facing the most challenging crisis since World War II (Associated Press, 2020). With the pandemic taking on an unprecedented magnitude in the twenty-first century, it quickly monopolized media attention. As of early April, Radar+'s large dataset showed that about 65 per cent of headlines on major Canadian media websites were related to the COVID-19 pandemic.

While the crisis has attracted some scholarly attention in Canada, few studies have yet focused on the coverage of the crisis and its evolution over time. However, media coverage is broadly recognized as having the potential to influence public attitudes (McCombs and Shaw, 1972). The audience perception of a crisis is often said to be shaped, or altered, by journalists. They can do so in multiple ways: by the level of attention devoted to an issue, by the tone in which an issue is discussed and by the frames through which an issue is presented (Damstra and Vliegenthart, 2018).

Given the global nature of the COVID-19 pandemic, the role of Canadian media in framing the crisis deserves special attention. Drawing on a large and unique dataset, we rely on automated textual analysis to address the following questions: *How do Canadian mainstream media frame the COVID-19 crisis in their reports? How do they differ in their use of these frames?* Topic modeling enables us to detect six main topics in our corpus of texts and allows us to grasp the contrasting differences in the coverage of the COVID-19 pandemic across media and over time.

## Framing Theory

Media have to be selective about what they present to their audiences—they are telling a story about the world despite the presence of factual elements in the stories (Gamson and Modigliani, 1989). This story-telling process is referred to as framing. According to Entman (1993), selection and salience are two key features of framing. He suggests that to frame is to “select some aspects of a perceived reality and make them more salient in a communicating text, in such a way as to promote a particular problem definition, causal interpretation, moral evaluation, and/or treatment recommendation” (Entman, 1993: 52). Frames are consequential: they lead the audience to interpret issues in various ways. When elevated in salience, a piece of information is more likely to be noticeable, meaningful, or memorable to audiences (Entman, 1993). Indeed, the framing process is an influential way through which the media may shape public opinion or alter citizens’ attitudes (Iyengar and Kinder, 1987). At the same time, people's processing of information is influenced by pre-existing meaning structures or attitudes. For instance, people who are generally poorly informed but cognitively active will be particularly receptive, and thus heavily influenced, by the framing process (Zaller, 1992). Previous research has identified various frames that occur commonly in the news, but very few studies have investigated which frames have been used in the coverage of crises (An and Gower, 2009). This research attempts to fill this gap and uses topic modelling to investigate the media framing of COVID-19 in the Canadian context.

## Data and Method

This study analyzes news articles related to the COVID-19 pandemic as covered by 12 major Canadian media sources.^1^ These media were chosen because they represent the online news sources with the highest average readership in Canada and in Quebec.^2^ Data are continuously collected using Radar+, a tool developed in Python for digital content extraction and automated text analysis.^3^ By making certain issues or attributes more salient in the news, media influence the importance attributed to these issues by mass audiences. Moreover, a significant number of citizens are only aware of issues that are very salient in the news (Iyengar, 2016). As such, “front page” stories—the article that is the most prominent on the homepage of each media outlet's website—were chosen as the unit of analysis. The final dataset contains 2,810 articles (francophone media = 968; anglophone media = 1842) for a total of 989,345 words.^4^

All front-page articles where the headline referred to the COVID-19^5^ crisis were derived from Radar+'s database, covering the period January 11–April 11, 2020. January 11 marks the first time an article about the COVID-19 pandemic made the front page in a major Canadian media outlet. Multiple rounds of pre-tests showed that loosening this criterion resulted in a subset containing some articles in which the crisis was only briefly mentioned.

We rely on latent Dirichlet allocation (LDA) with Gibbs sampling for topic modeling, using the R package *topicmodels*. LDA is an unsupervised machine learning method, which means that the researcher gives no input as to how the data should be classified. To ensure objectivity, previous framing analyses have privileged this inductive approach (Tian and Stewart, 2005). LDA assumes that each document is an assortment of topics (DiMaggio et al., 2013). Topics are essentially probability distributions over a corpus of words (Blei et al., 2003).

Recent research suggests that LDA is an appropriate tool for analyzing news media coverage (Daud et al., 2010), since topics can be interpreted as frames (Ylä-Anttila et al., 2018). The only input given by the researchers, in addition to the data itself, is the number of topics. Different numbers of topics were tried: too many resulted in the over-clustering of the corpus into many highly similar topics, while the contrary resulted in topics that were too broad. Although the French and English models were run separately, the appropriate number of topics for each corpus was six. Frames were validated and interpreted by five researchers through a reflexive process, and tentative, descriptive names were given to each frame using the top 15 words of each topic.^6^ The resulting frames were the following: *Chinese Outbreak*, *Economic Crisis*, *Western Deterioration*^7^, *Health Crisis*, *Social Impact* and *Helping Canadians.*^8^

## Results

**Figure 1.**
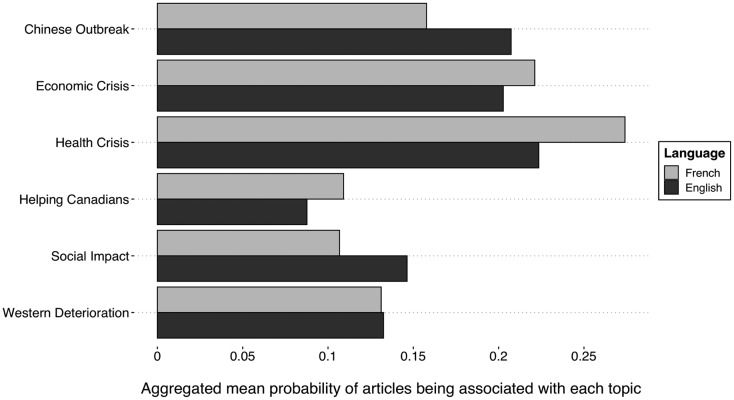
Aggregated mean of topic usage.

Source: Radar+, January 11–April 11, 2020.

Note: n = 2,810.

Figure 1 shows the aggregated mean probability of articles associated with each frame per corpus language.^9^ The most common frame for both corpora is *Health Crisis*. This figure also shows that, overall, the francophone media framed the COVID-19 pandemic more as an emergency to help Canadians abroad and as an *Economic Crisis*, while the anglophone media used the *Chinese Outbreak* and the *Social Impact* frames more frequently. These differences in means were found to be statistically significant.^10^

**Figure 2.**
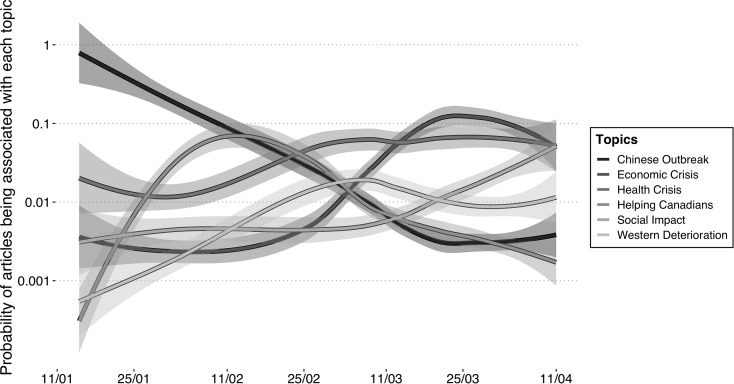
Longitudinal topic evolution of francophone COVID-19 headline coverage.

Source: Radar+, January 11–April 11, 2020.

Note: n = 968.

**Figure 3.**
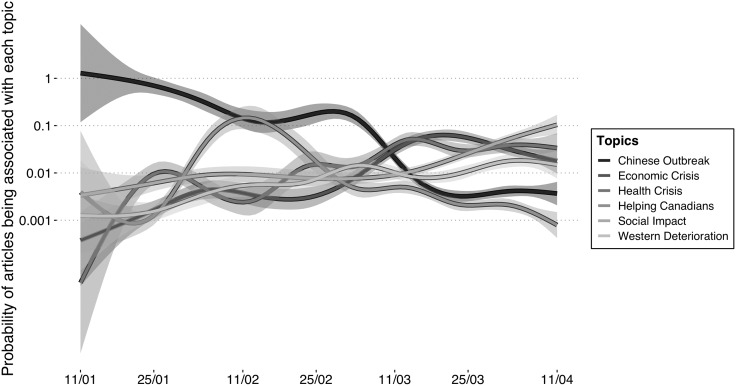
Longitudinal topic evolution of anglophone COVID-19 headline coverage.

Source: Radar+, January 11–April 11, 2020.

Note: n = 1,842.

Figures 2 and 3 show that both francophone and anglophone media coverage began with an emphasis on the Chinese outbreak. The lines display smoothed conditional means, and the shaded areas represent the standard error. As the crisis evolved in Canada, it was framed more as both a health and an economic crisis. These figures also indicate that framing of the crisis as *Helping Canadians* abroad reached its height in February. We find discrepancies in the degree to which each frame was being used between francophone and anglophone media, but no differences in tendencies. Only at the end of the period do the anglophone media distinguish themselves by making *Social Impact* the most important frame.

**Figure 4.**
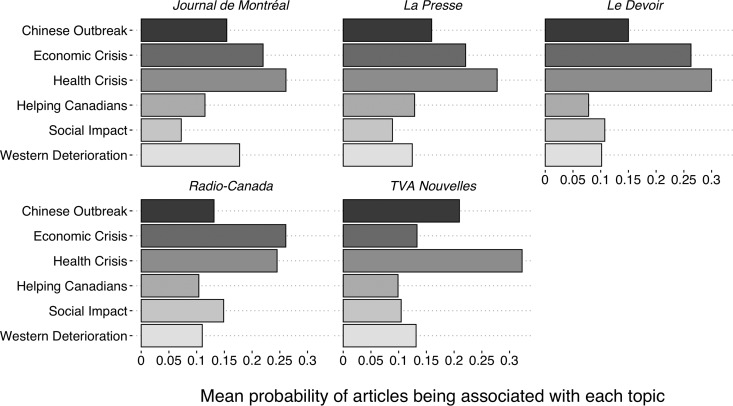
Mean topic used by each francophone media outlet.

Source: Radar+, January 11–April 11, 2020.

Note: n = 968.

**Figure 5.**
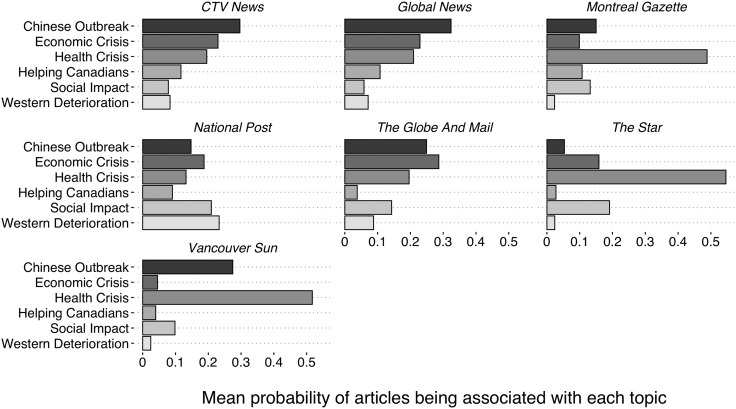
Mean topic used by each anglophone media outlet.

Source: Radar+, January 11–April 11, 2020.

Note: n = 1,842.

Figures 4 and 5 break up the results shown in Figure 1 by media. For the francophone media, Radio-Canada and TVA Nouvelles stand out. Radio-Canada is the only francophone media that favours—although slightly—the *Economic Crisis* frame the most, while TVA Nouvelles framed the COVID-19 crisis mostly as a *Health Crisis*. The francophone disparities are marginal compared to the anglophone ones. CTV News and Global News offer an almost identical use of frames, with the *Chinese Outbreak* being the most important. *The Star* framed the COVID-19 pandemic mostly as a *Health Crisis*. *The Montreal Gazette* and the *Vancouver Sun* offer a similar coverage as *The Star*, but with a more important emphasis on the *Chinese Outbreak* for the latter. The most distinctive coverage comes from the *National Post* and *The Globe and Mail*. In fact, the *National Post* is the only media that favors the *Western Deterioration* frame on its front page. *The Globe and Mail* is the only anglophone media that mostly framed the COVID-19 pandemic as an *Economic Crisis*.

## Discussion

This article set out to examine the framing of the COVID-19 pandemic by empirically analyzing the front pages of 12 well-known news media outlets. Using a machine learning method, LDA, the same frames were identified for both the anglophone and francophone media. Canadian media covered the crisis extensively, with the *Health Crisis* frame being the most frequently used. This is not surprising, considering that, as of early April, coronavirus-related deaths reached 100,000 worldwide (Breen, 2020).

We find a noticeable difference in the use of the *Health Crisis*, *Social Impact* and *Chinese Outbreak* frames between francophone and anglophone media. Our results suggest that the disparity in received information is more substantial among anglophone mass audiences than among the francophone ones. Although we live in a global information era, there are still within-country differences in the coverage of a single news story.

Finally, this study also suggests that topic modeling can be a useful approach to frame analysis. Its inductive component also allows for a more objective interpretation of the meaning of texts than the traditional manual text analysis with prespecified frames (Tian and Stewart, 2005). Hopefully quantitative and qualitative approaches to frame analysis can be integrated into a coherent framework for future research.

## Notes

1 Radio-Canada, *Le Journal de Montréal*, *Le Devoir*, *La Presse*, CTV News, *Montreal Gazette*, Global News, *The Globe and Mail*, *Vancouver Sun*, *National Post*, and *The Star*. There were no articles collected from CBC News due to a technical error. For the same reason, no articles were collected from the *Vancouver Sun* since March, which explains the small sample size (see online Appendix). One needs to keep this in mind when interpreting the results.

2 We kept the largest media outlets in Quebec in terms of audience to allow for a comparison between francophone and anglophone media.

3 Radar+ extracts front-page content from 12 Canadian media outlets every five minutes and saves it to a database. For more information: https://www.clessn.com/radar/index.html.

4 Stopwords, punctuation and numbers were removed using the R package *tm*. See Figures A1 and A2 (available on the online Appendix) for the longitudinal distribution of words per day for each media.

5 See online Appendix for more information.

6 See Table A1 in the online Appendix.

7 By which we mean the deterioration of the social and health conditions in countries like Italy, France and the United States.

8 By which we mean the federal effort to safely bring back Canadians overseas.

9 A two sample Kolmogorov–Smirnov test was used to measure if the topic distributions between francophone and anglophone media differ statistically (see Table A4 in the online Appendix).

10 See Table A5 in the online Appendix.

## Supplementary material

For supplementary material accompanying this paper visit http://dx.doi.org/10.1017/S0008423920000372. click here to view supplementary material
