# Supplementary material for: (Un)Covering the COVID-19 Pandemic: Framing Analysis of the Crisis in Canada
Source: Can J Polit Sci. 2020 Apr 29:1–7. doi: 10.1017/S0008423920000372 (PMC7256214; doi:10.1017/S0008423920000372)
Supplement: Supplementary file 1 [file S0008423920000372sup001.docx]

(Un)covering the COVID-19 pandemic

Framing analysis of the crisis in Canada

# Online Appendix


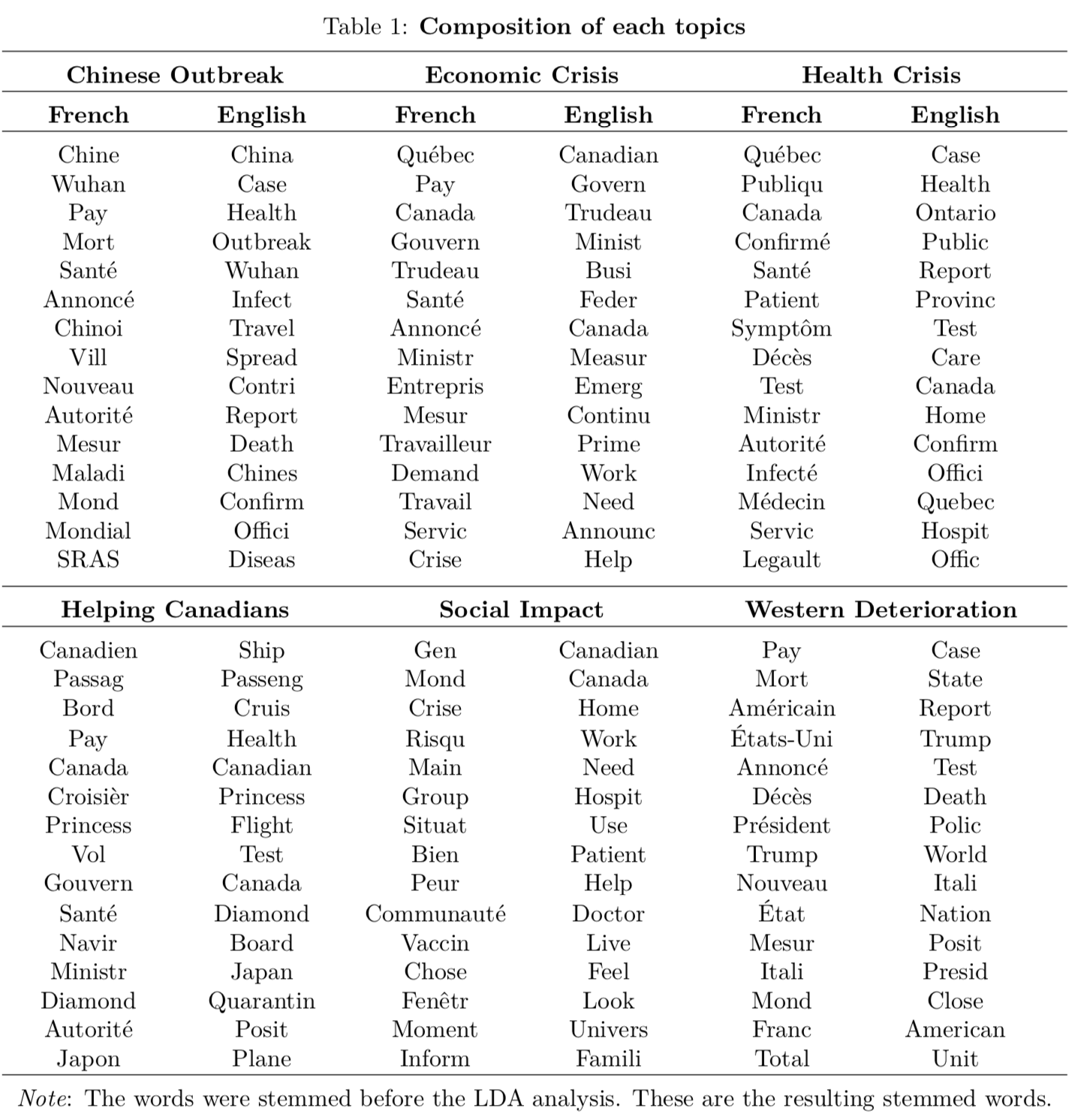


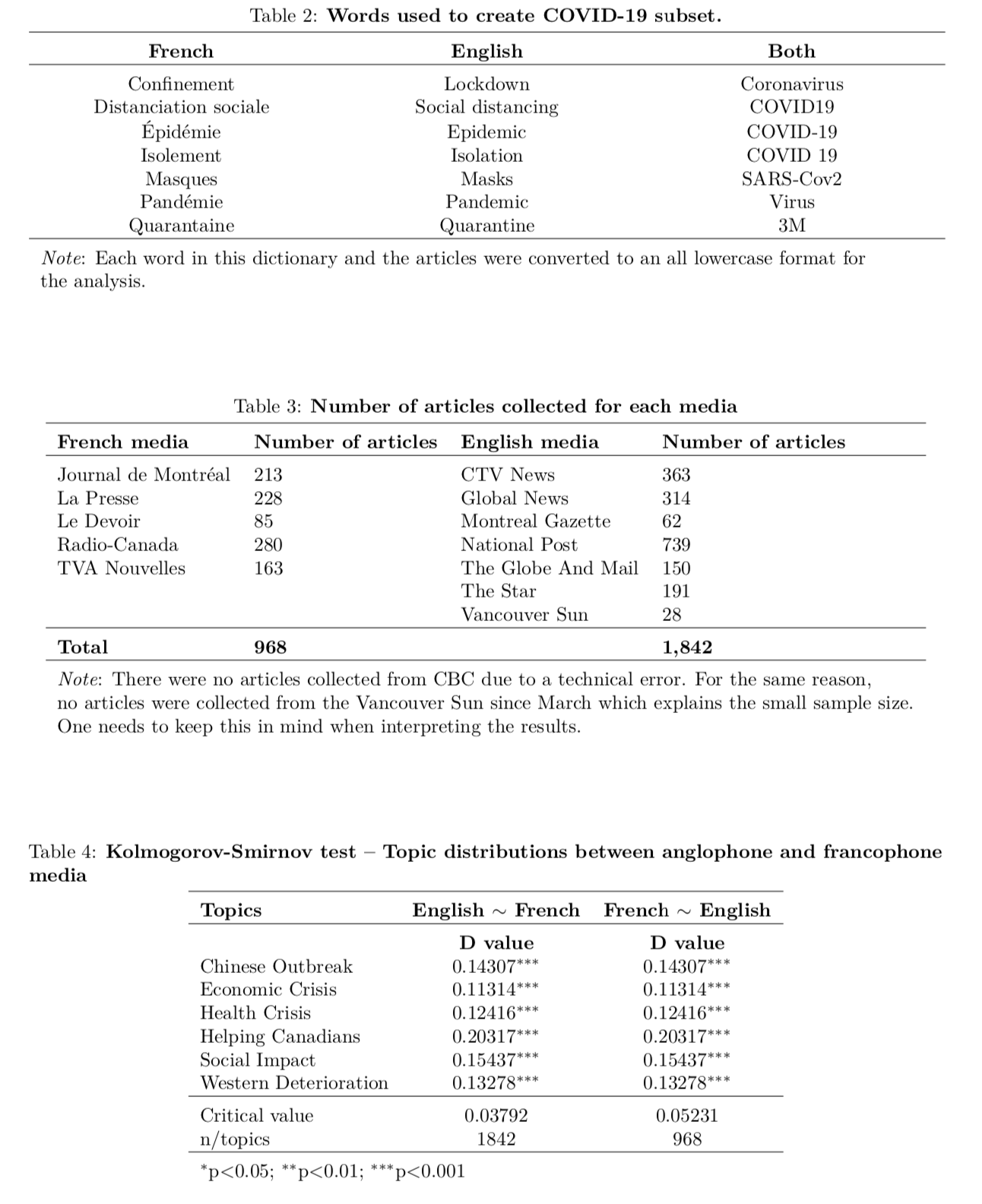


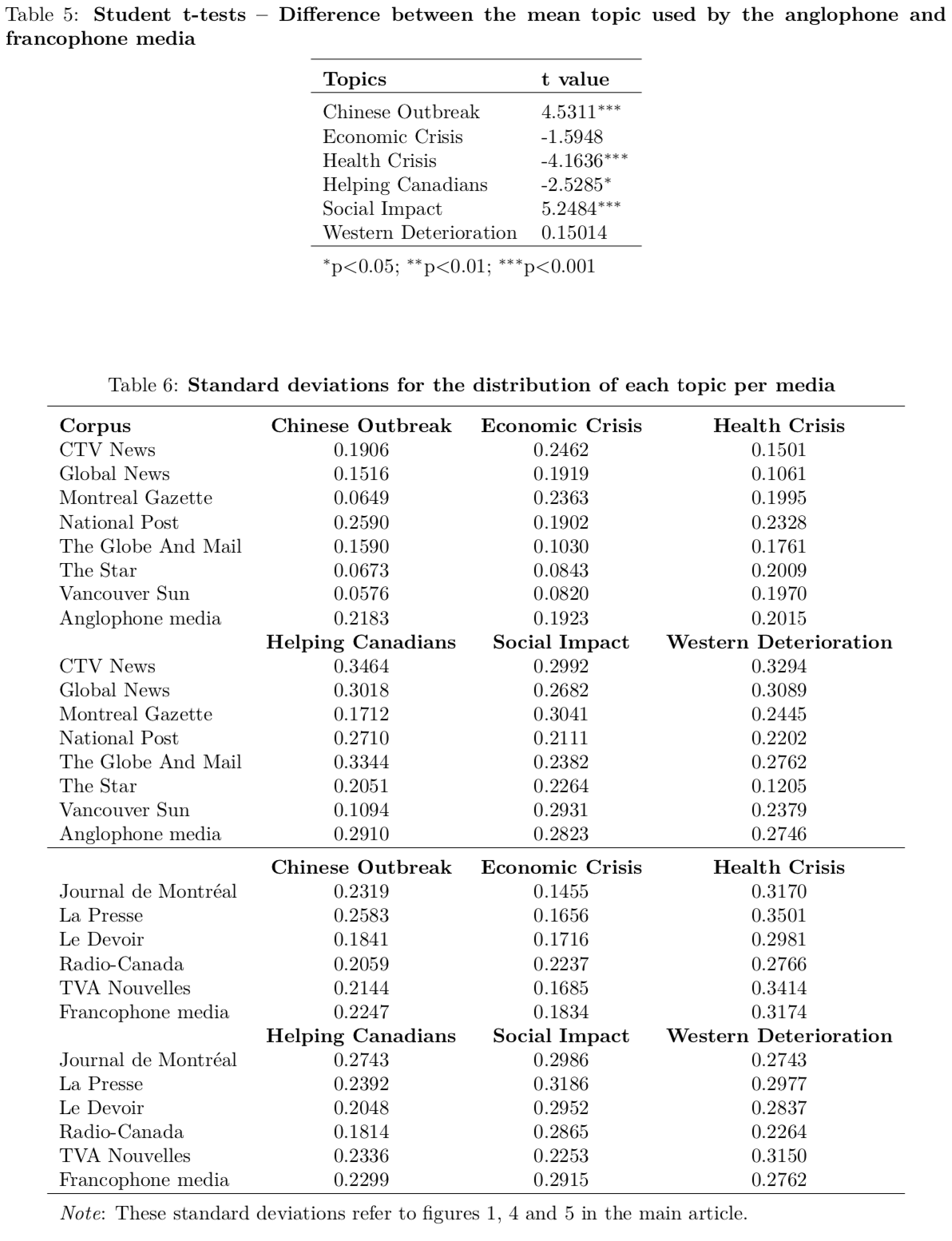


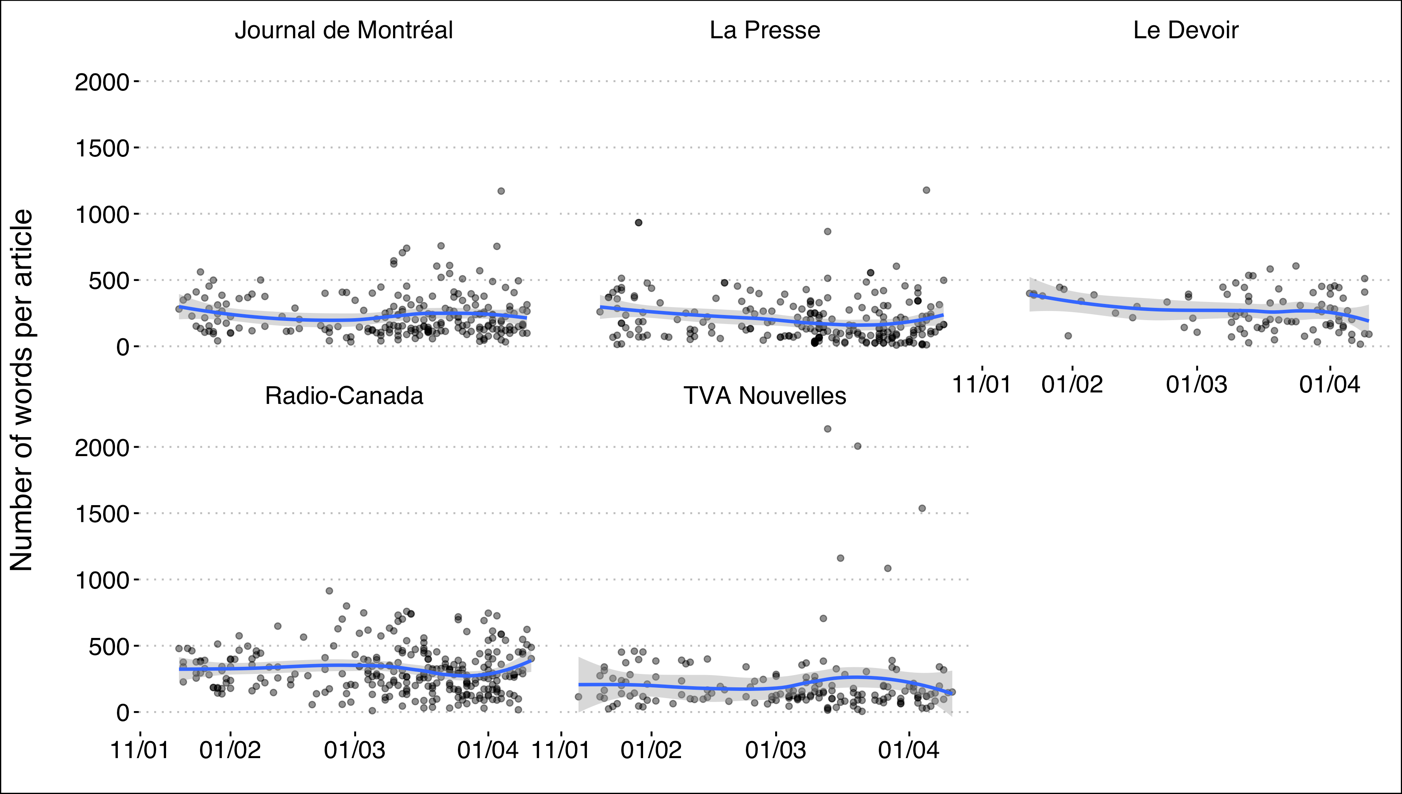


**Figure A1**: Number of words per article per day for each francophone media

Source: Radar+, from January 11^th^ to April 11^th^, 2020.

Note: n = 968.


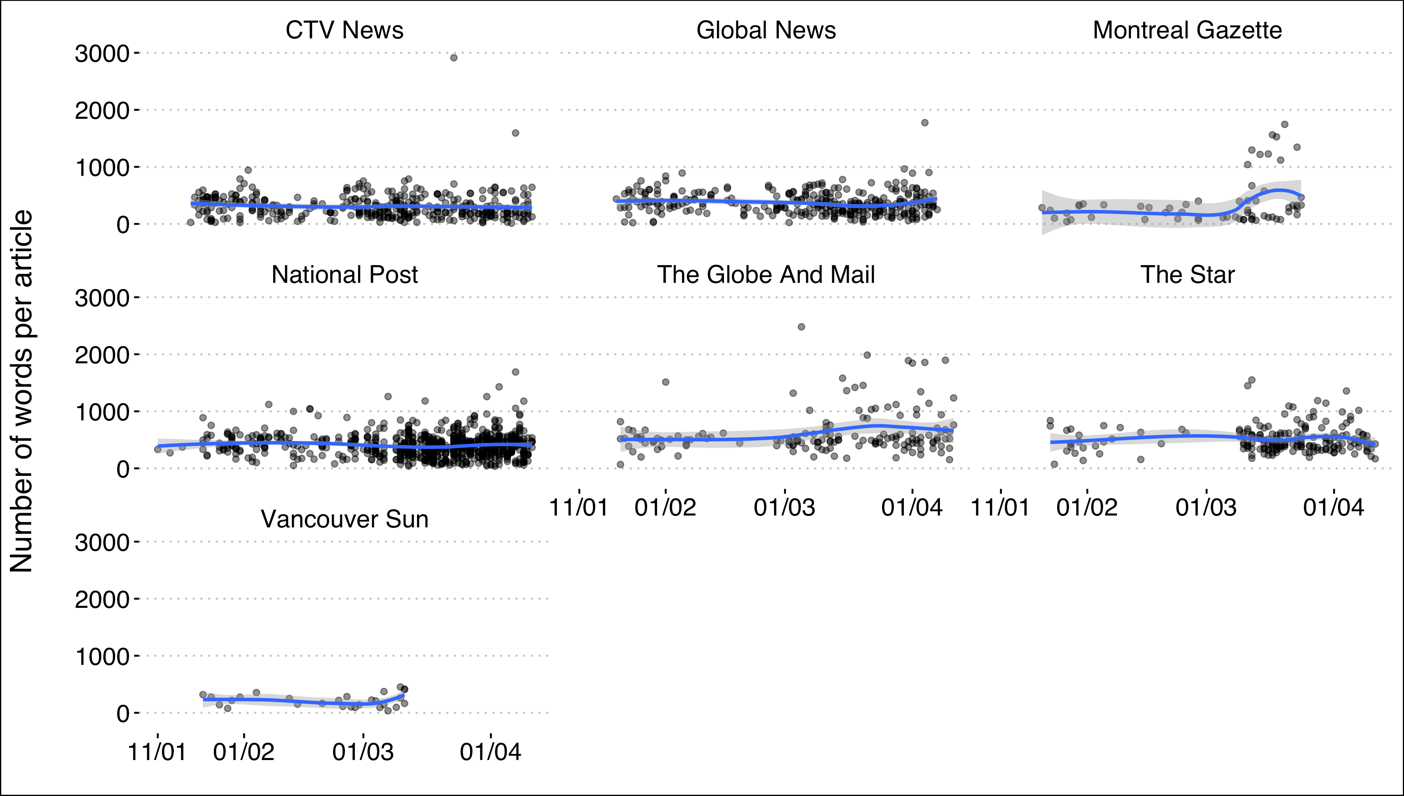


**Figure A2**: Number of words per article per day for each anglophone media

Source: Radar+, from January 11^th^ to April 11^th^, 2020.

Note: n = 1,842.
